# Which is the current knowledge on man-made endocrine- disrupting chemicals in follicular fluid? An overview of effects on ovarian function and reproductive health

**DOI:** 10.3389/fendo.2024.1435121

**Published:** 2024-10-02

**Authors:** Anna-Mariia Shulhai, Valentina Bianco, Valentina Donini, Susanna Esposito, Maria Elisabeth Street

**Affiliations:** ^1^ Department of Medicine and Surgery, University of Parma, Parma, Italy; ^2^ Department of Pediatrics №2, Ivan Horbachevsky Ternopil National Medical University, Ternopil, Ukraine; ^3^ Unit of Paediatrics, University Hospital of Parma, P. Barilla Children’s Hospital, Parma, Italy

**Keywords:** endocrine-disrupting chemicals, EDCs, follicular fluid, female fertility, ovary, oocytes

## Abstract

The increase in female reproductive disorders, such as polycystic ovary syndrome, endometriosis, and diminished ovarian reserve that lead to subfertility and infertility, has encouraged researchers to search and discover their underlying causes and risk factors. One of the crucial factors that may influence the increasing number of reproductive issues is environmental pollution, particularly exposure to man-made endocrine-disrupting chemicals (EDCs). EDCs can interfere with the ovarian microenvironment, impacting not only granulosa cell function but also other surrounding ovarian cells and follicular fluid (FF), which all play essential roles for oocyte development, maturation, and overall reproductive function. FF surrounds developing oocytes within an ovarian follicle and represents a dynamic milieu. EDCs are usually found in biological fluids, and FF is therefore of interest in this respect. This narrative review examines the current knowledge on specific classes of EDCs, including industrial chemicals, pesticides, and plasticizers, and their known effects on hormonal signaling pathways, gene expression, mitochondrial function, oxidative stress induction, and inflammation in FF. We describe the impact of EDCs on the development of reproductive disorders, oocyte quality, menstrual cycle regulation, and their effect on assisted reproductive technique outcomes. The potential transgenerational effects of EDCs on offspring through animal and first-human studies has been considered also. While significant progress has been made, the current understanding of EDCs’ effects on ovarian function, particularly in humans, remains limited, underscoring the need for further research to clarify actions and effects of EDCs in the ovary.

## 1 Introduction

A decrease in fertility rates and an increasing number of reproductive disorders are reported worldwide (1). According to the World Health Organization, infertility affects almost one out of every six people (2). Studies have shown that disorders in women such as endometriosis and polycystic ovary syndrome (PCOS) have a crucial impact on ovarian function and fertility; both have presented an alarming increase in the last decade (3). Furthermore, factors such as advanced maternal age, high body mass index, bad habits, and environmental exposure to pollutants, like endocrine-disrupting chemicals (EDCs), contribute to the modification of oocyte quality, changes in follicular fluid, and as a result, ovarian disorders, subfertility, and infertility (4).

One of the environmental factors that has been increasing with globalization in the last 50 years is exposure to EDCs (5, 6). EDCs are synthetic and/or natural chemicals that interfere with the body’s endocrine system, including ovaries. These chemicals can mimic hormones, disrupt hormonal signaling, block hormone receptors, or interfere with hormone synthesis, leading to a variety of adverse effects on reproductive health. They can be found in everyday products, such as personal care items, cosmetics, plastics, detergents and pesticides, electronics, building materials, etc. Exposure to EDCs can occur by ingestion, inhalation, and skin contact spreading to many organs in the human body through fluids, including ovaries, and placenta (5–7). Some studies have suggested that exposure to EDCs during critical periods of ovarian development, such as fetal development or puberty, can have long-lasting effects on ovarian function and reproductive health (8). There is scientific evidence that exposure to EDCs, such as bisphenol A (BPA), phthalates, and certain pesticides, can disrupt hormonal signaling, leading to irregularities in the menstrual cycle, fertility issues, and even to an increased risk of conditions such as polycystic ovary syndrome or ovarian cancer (9, 10). EDCs are usually found and easily assayed in human fluids such as urine, serum, and breast milk and in some tissues (mainly lysates), and cells (5–7). Some studies describe the impact of bisphenols, phthalates, and pesticides on oocyte and granulosa cells (11–14). However, there are only a few studies that have addressed the presence and composition of EDCs in follicular fluid.

Follicular fluid (FF) is a complex biological fluid surrounding the oocyte within the ovarian follicle and plays a supportive role in creating its microenvironment, supporting normal folliculogenesis and germ cell-somatic cell communication. It is secreted by granulosa cells in antral follicles and serves as a reservoir containing a diverse array of proteins, amino acids, lipids, hormones, growth factors, antioxidants, and metabolites essential for several cellular processes as protein synthesis, DNA replication, and mitochondrial function which are key for good fertilization and embryo development. Among the hormones contained in FF are estradiol, progesterone, human chorionic gonadotropin (hCG), follicle-stimulating hormone (FSH), luteinizing hormone (LH), growth hormone (GH), and insulin-like growth factors (IGF) system peptides. In addition, FF contains antioxidants such as superoxide dismutase and glutathione that help neutralize reactive oxygen species (ROS) generated during oocyte maturation. Maintaining optimal antioxidant levels in the follicular microenvironment is crucial for protecting oocytes from oxidative stress-induced damage and preserving their developmental competence. Growth factors such as epidermal growth factor (EGF), and vascular endothelial growth factor (VEGF) are also found in FF, and are important for cell proliferation, differentiation, angiogenesis, and tissue remodeling within the ovarian follicle (15, 16). Finally, FF contains high mobility group box 1 (HMGB1), which is a protein with cytokine activity associated with PCOS in women (17). Moreover, it contains extracellular vesicles containing cell-free RNA, particularly miRNAs (16, 17). The composition of FF thus mirrors the metabolic and hormonal state of the developing follicle and serves as a valuable substrate to assess oocyte quality. Consequently, studying FF provides insights that can optimize assisted reproductive technologies outcomes and enhance understanding of reproductive disorders (15, 18, 19). Therefore, EDCs exposure may contribute to the development of reproductive diseases.

The aim of this review is to explore current knowledge and the potential impact of EDCs in ovaries with a specific focus on FF. We discuss their mechanisms of action within the ovarian microenvironment, and the possible effects on oocyte quality, hormonal regulation, and reproductive outcomes.

## 2 Methods

The literature was researched using specific research strings in Pubmed, Scopus, and Mendeley via MeSH using specific keywords. The WHO and the European international official sites were also considered. The identified records were imported into Mendeley. After removing duplicates, reviewers separately screened the included papers found through the literature search by title and abstract, using the eligibility criteria, published from 1994 to March 2024. The following keywords were used for the search: endocrine-disrupting chemicals, endocrine disruptors, EDCs, bisphenol, phthalates, pesticides, dioxin, follicular fluid, ovaries, and reproductive health, which were differently matched for all required strings. A narrative synthesis was conducted to organize the search findings and summarize current knowledge to disclose knowledge gaps.

## 3 Mechanism of action of EDCs in follicular fluid and ovaries

**Figure 1:**
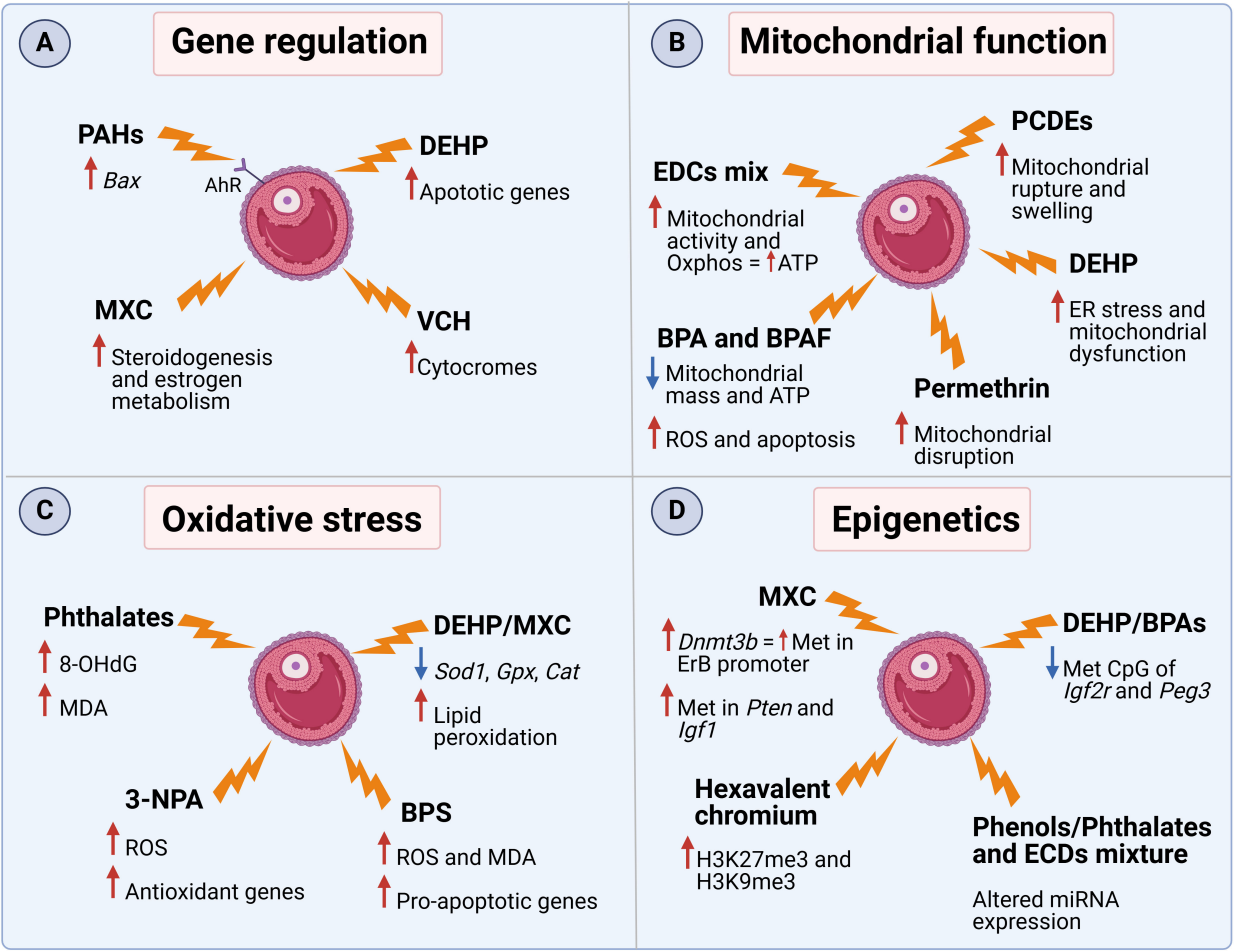
The mechanism of action of several EDCs in FF and ovaries. Molecular effects related to **(A)** gene expression, **(B)** mitochondrial function, **(C)** oxidative stress, and **(D)** epigenetics. PAHs (polycyclic aromatic hydrocarbons), DEHP (diethylhexyl phthalate), MXC (methoxychlor), VCH (4-vinylcyclohexene), PCDEs (polychlorinated diphenyl ethers), BPA (bisphenol A), 3-NPA (3-nitropropionic acid), BPS (bisphenol S). This Figure was created with BioRender.com.

Despite the limited number of studies on FF, some studies identified pesticides, industrial lubricants and solvents, phthalates, and flame retardants as the main EDCs present in ovaries and, particulary, follicular fluid (**Table 1**) (19–22). Most of these EDCs are able to bind to serum proteins such as albumin for efficient transport in the circulation (23, 24). Since albumin can cross the blood follicle barrier, it can be hypothesized that EDCs are readily transferred from the bloodstream into the developing follicle, where they exert their negative effects. As EDCs can affect initial and late phases of folliculogenesis, deplete the primordial follicle reserve through atresia or premature activation, reducing the number of follicles available for ovulation, inhibit FSH-dependent maturation, production of estrogen and LH (7, 25). However, most research describes the molecular effect of EDCs in ovaries, without considering their specific impact in FF. Therefore, in the following sections, we highlight relevant literature describing the numerous interconnected molecular mechanism that EDCs trigger mainly in the ovaries and partly in FF (**Figure 1**).

**Table 1:** Common endocrine-disrupting chemicals found in follicular fluid and their main sources.

EDCs	Route of exposure	Sources	References
Bisphenols	Ingestion Dermal Inhalation* Transplacental	Polycarbonate plastic (re-usable plastic tableware, water bottles, sports equipment), epoxy resin (food and beverage cans, coat the insides of water pipes, flooring, car body coatings), thermal paper, inks, textiles.	(5, 6, 10)
Phthalates	Ingestion Dermal Inhalation Transplacental*	Contaminated food products, plastic containers, vinyl flooring, lubricating oils, and personal-care products, like soaps, shampoos, hair sprays, perfumes, cosmetics.	
Per- and polyfluoroalkyl substances (PFAS)	Ingestion Inhalation Transplacental* Dermal*	Contaminated food products (fish, meat, water), manufacturing facilities, aqueous film-forming foam applications, nonstick cookware (teflon), metal coating operations, textile and paper coating, personal-care products, like shampoos, dental floss, nail polish.	
Pesticides	Dermal Ingestion Inhalation Transplacental*	Contaminated food products by agricultural pesticides herbicides, contaminated water by leaking storage tanks, rainwater runoff from over-treated areas, spray drift, and improper disposal of pesticides agricultural herbicides.	
Dioxins	Ingestion Dermal Inhalation	Contaminated food products (meat and dairy products, fish and shellfish), cigarette smoke, chlorine bleaching of paper pulp, metals smelting, volcanic eruptions, combustion of wood, coal, oil.	
Polychlorinated biphenyls (PCBs)	Ingestion Inhalation Dermal Transplacental*	Industrial solvents or lubricants for equipment (pumps, compressors, conveyors, hydraulic systems, and turbines), paints, oil, cable insulation.	
Flame retardants	Inhalation Ingestion Dermal	Insulation foams, foam of mattresses, carpets, coverings of cars, foam of mattresses, carpets, electronics devices, electrical cables, plugs adhesives.	

*There are only limited studies on some metabolites and a small amount can be transferred through this route.

### 3.1 Disruption of hormonal signaling pathways and changes in gene expression

As the structure of some EDCs’ is similar to that of endogenous steroid hormones, they are able to bind to aryl hydrocarbon receptors (AhR) or estrogen receptors (ERs), stimulating the secretion of 17β-estradiol (E2) (26). For example, it was demonstrated that a mix of EDCs, consisting of PFOA, PFOS, HCB, p,p’-DDE, and PCB153, was able to increase E2 secretion via both classical and non-classical ER pathways in a human granulosa tumor cell line (27). Furthermore, treatment of murine antral follicles with the pesticide methoxychlor (MXC) resulted in decreased expression of several steroidogenic enzymes as well as the estrogen metabolic enzyme Cyp1b1, resulting in diminished sex steroid hormone levels (28). Moreover, long-term exposure of granulosa cells to an EDCs mix (BPA [2 ng/mL], PCB 153 [70 pg/mL], B[a]P [1 ng/mL], and PFOS [100 pg/mL]) led to a reduction in the protein level of the key enzyme for estradiol synthesis, aromatase CYP19A1, with a subsequent decrease in estradiol production after 2 weeks of exposure (13). In addition to steroid synthesis, EDCs can influence other aspects of cell homeostasis. Activation of AhR by polycyclic aromatic hydrocarbons (PAHs), contaminants released via incomplete organic fuel combustion, drive the over-expression of Bax, a pro-apoptotic gene, resulting in accelerated oocyte depletion in female mice (29, 30). Similarly, exposure to diethylhexyl phthalate (DEHP), a plastic additive, increased the expression of several apoptotic genes in pre-pubertal murine ovaries (31). Another major contributor to ovotoxicity is 4-Vinylcyclohexene (VCH), a compound found in cigarette smoke. It was demonstrated that VCH administration in mice increases the expression of several ovarian cytochrome isoforms, which are responsible for the conversion of VCH into its more toxic metabolite, responsible for destruction of immature, small preantral follicles (32).

### 3.2 Interference with mitochondrial function

Cholesterol shuttling to mitochondria is essential for steroid hormone synthesis (33, 34). Polychlorinated diphenyl ethers (PCDEs), found mainly in aquatic samples, have been reported to interfere with many cellular organelles in Zebrafish ovarian cells, such as the endoplasmic reticulum (ER) and mitochondria, causing ruptures and swelling (35). Likewise, in rats treated with the pesticide permethrin, a negative effect of EDCs was observed on ovarian mitochondria morphology, resulting in organelle disruption (36). Another study involving the treatment of human granulosa cells with a mixture of several EDCs (PFOA, PFOS, HCB, p,p’-DDE, and PCB153) showed changes in mitochondrial shape associated with increased activity with a shift toward oxidative phosphorylation, that translated into increased ATP content (37). In addition, the co-exposure to BPA and bisphenol AF (BPAF) in human granulosa cells revealed reduced mitochondrial mass and ATP production, damage to the mitochondrial respiratory chain, and increased ROS production with consequent induction of apoptosis mechanisms (38). Finally, female mice exposed to DEHP showed ER stress and mitochondrial dysfunction with reduction of membrane potential and induction of apoptosis (39). It was then demonstrated that these changes were due to modifications in the structure of the ER membranes and the suppression of oxidative phosphorylation pathways (39). These data suggest that the altered mitochondrial morphology and function might be the cause of impaired steroid production and increased follicular atresia after EDC exposure.

### 3.3 Induction of oxidative stress and inflammation

An additional mechanism that EDCs exploit to interfere with ovarian functions is activating ROS production. For example, exposure to DEHP or MXC was able to decrease the expression of *Sod1*, *Gpx* and *Cat*, key antioxidant enzymes, in murine ovarian somatic cells and antral follicles respectively (31, 40). The production of ROS after exposure to MXC was associated with a higher rate of lipid peroxidation, resulting in increased ovarian toxicity in rats (41). In addition, the presence of several phthalate metabolites in the FF of women undergoing *in vitro* fertilization was positively associated with 8-hydroxy-2′-deoxyguanosine (8-OHdG), an oxidized derivative of deoxyguanosine, and malondialdehyde (MDA), both used as oxidative stress biomarkers (42). Accumulation of MDA and ROS were also found in the offspring of BPS-treated ICR female mice, accompanied by increased expression levels of pro-apoptotic markers (Bax, Caspase 3, and Caspase 9) and decreased expression levels of anti-apoptotic marker (Bcl2) (43). In addition, BPS also activated the mTOR pathway, subsequently down-regulating autophagy (43). Similarly, also rats injected with atrazine, a common herbicide, showed increased ovarian levels of MDA, reduction in the activity of ovarian GST and SOD enzymes, higher ovarian expression of inflammatory cytokines and pro-apoptoric markers (44). Furthermore, exposure to 3-nitropropionic acid (3-NPA), a mycotoxin, increased ROS concentration in granulosa cells, despite the increase in the aforementioned antioxidant genes (45).

### 3.4 Epigenetic regulation

A third way that EDCs exploit to disrupt ovarian homeostasis is the modulation through epigenetic modifications. It was shown that DEHP or BPA exposure was able to reduce the number of methylated CpG sites of Igf2r and Peg3 in murine germ cells (46, 47). These genes are representatives of imprinted genes during gametogenesis and the modification was also maintained in the offspring. In addition, temporary exposure to MXC during the development of the rat fetus and newborn led to higher expression of Dnmt3b, a DNA methyltransferase, that causes increased methylation of the ERβ promoter regions (48). Furthermore, exposure to hexavalent chromium in rats increased H3K27me3 and H3K9me3, heterochromatin-associated repressive histone markers, whereas decreased H3K9ac and H3K27ac, acetylation marks in histones H3 (49). A more recent study suggested that other genes are affected by MXC-induced methylation. Key pathways such as PTEN signaling, IGF-1 signaling, or rapid estrogen signaling were hypermethylated and, therefore, suppressed, resulting in dysregulated early folliculogenesis as well as follicular maturation and ovulation in rats (50). Furthermore, a recent publication associated urine metabolites of several EDCs with changes in the expression of miRNAs related to follicular development and oocyte competence (51). Finally, a follow up study assessed the correlation between EDC concentrations in FF and miRNA changes. It was observed that the expression of 39 miRNAs correlated with MEP, MCOMOP, MCOMHP, MBzP and/or with mECPP content (52). Interestingly, these miRNAs were associated with pathways involved in oocyte development, oocyte maturation and fertilization, suggesting that miRNAs play an important role in regulating female fertility (52).

In summary, the above mentioned studies provide valuable insights into the mechanisms underlying EDC-induced ovarian dysfunction, there remains a notable gap in our understanding of how concurrent exposure to multiple EDCs may exacerbate adverse outcomes. Future investigations incorporating the complexities of EDC mixtures are essential for a comprehensive assessment of their impact on ovarian health.

## 4 Effects of EDCs on oocyte quality and development

### 4.1 Implications for ovulation, PCOS, fertility and endometriosis

Exposure to EDCs can cause changes in the female reproductive system. Higher and persistent exposure to EDCs can modify the follicular micro-environment, affect oocyte quality, and may trigger disease development, such as PCOS, endometriosis, fertility and ovulation disorders (7, 20, 53).

PCOS is clinically characterized by hormonal changes, as hyperandrogenism, chronic oligo/anovulation, characteristic ovarian morphology on gynecological ultrasound, increased anti‐Müllerian hormone (AMH) levels, and metabolic disorders such as obesity, insulin resistance, and atherogenic dyslipidemia. The etiology of PCOS is not fully understood, but potential contributing factors may include exposure to high levels of AMH, androgens and EDCs during the prenatal period (54–56). For example, a significant correlation was described between BPA and PCOS (56, 57). BPA is a xenoestrogen that mimics the activity of 17-β-estradiol and can block its feedback pathway at the hypothalamic-pituitary and ovarian levels, causing inadequate hormone production, menstrual cycle abnormalities, infertility and impaired development and functioning of the reproductive system (55, 58). Dysregulation of gene expression in the hypothalamic-pituitary region caused by BPA exposure can lead to an excessive release of GnRH (59). This, in turn, induces a continuous increase in LH and a reduction in FSH secretion. The disrupted FSH/LH balance impairs ovarian follicle development, while the elevated LH levels contribute to increased ovarian androgen production (60, 61). Additionally, BPA may alter the gene expression of ovarian steroidogenesis genes, such as 17α-hydroxylase, downregulating estradiol synthesis and contributing to hyperandrogenism and ovulatory dysfunction (62). In addition, another recent study observed an increased concentration of BPA in FF in women with PCOS (63). This dose-dependent increase in the EDC was associated with a downregulation of aromatase, a key enzyme in the aromatization of androgens into estrogens, in granulosa cells, leading to reduced E2 synthesis and hyperandrogenism (63).

Similarly, other studies have shown that exposure to PFAS and DEHP is associated with clinical features of PCOS, such as irregular menstrual cycles or polycystic ovaries (64–66). A study comparing women with PCOS undergoing *in-vitro* fertilization (IVF) to infertile women without PCOS found that the former had significantly higher concentrations of DEPH in FF, leading to lower clinical pregnancy rate after IVF. Cell culture studies revealed that *in vitro* DEHP treatment on primary-cultured human granulosa cells and a steroidogenic human granulosa-like tumor cell line resulted in altered steroid production, decreased cell viability and proliferation, and increased apoptosis (57). This association is likely due to the action of the DEHP metabolite, mono(2-ethylhexyl) phthalate (MEHP), which acts on granulosa cells suppressing estradiol production, leading to anovulation (64, 65).

Furthermore, several studies have investigated the potential link between exposure to environmental pollutants and the development of endometriosis, a gynecological condition characterized by the presence of endometrial-like tissue outside the uterus. Studies have shown a positive correlation between endometriosis and exposure to organochlorine pesticides such as DDT and chlordane (67, 68), lead (69), and specific polychlorinated biphenyls (PCBs) (68, 70).

EDCs, as bisphenols and pesticides (DDE, DDT), may promote the crosstalk between cancer cells and surrounding stromal cells, increasing tumor development and invasion by influencing tumor microenvironment via mechanisms such as enhanced aromatase expression and growth factor release that activate estrogen-related pathways (71). Moreover, they can affect tumor growth by targeting protein Receptor for Activated C Kinase 1 (RACK1), which is the bridge between cancer progression and the immune system. EDCs modulate RACK1 expression thus promoting tumor growth by enhancing cell cycle progression and apoptosis regulation BPA has been shown to activate signaling pathways that facilitate cancer cell migration by increasing RACK1’s interaction with focal adhesion kinase, a key protein involved in cell adhesion and migration. Conversely, DDT may downregulate RACK1 expression resulting in decreased immunological responses and reduced production of pro-inflammatory cytokines (71).

### 4.2 EDCs and assisted reproductive technologies (ART)

The prevalence of reproductive health problems is increasing worldwide, together with assisted reproductive techniques (ARTs) (72). Despite the increased use of ART, the Assisted Reproductive Technology Surveillance report carried out in the United States in 2016 showed that success rates of live births remained similar (73). Several studies have investigated the link between EDCs and quality of embryos (20, 21, 74–76) and pregnancy rates (77–80). However, these studies were usually monocentric and failed to distinguish between factors affecting the mother’s ability to conceive and the impact on the father’s sperm health. Furthermore, these studies often examined the effects of individual EDCs, whereas real-world exposure typically involves a complex mixture of these chemicals. To address these limitations, a recent multicenter epidemiological study (81) investigated the link between EDCs and female fertility in medically assisted conception. The authors observed an inverse correlation of phthalates (82), DEHP metabolites and methylparaben, or a mixture of the two, with ovarian reserve (83, 84).

Supporting the notion that EDC mixtures might have a stronger impact on female fertility than single EDCs, a case-control study by Tian et al. investigated the effects of a mixture of 21 environmental contaminants (parabens, phenol, phthalates, and PFAS) found in follicular fluid of women who underwent IVF or intracytoplasmic sperm injection therapy. The study found that mixed and single EDCs were associated with an increased risk of diminished ovarian reserve. Moreover, EDC mixtures had a potentiating effect, meaning that the combined effect on reducing ovarian reserve was greater than the effect of each individual EDC alone. This reduction in ovarian reserve was particularly evident in women under 30 years of age (84).

Exposure to EDCs, which has also been linked to an increased risk of miscarriage. Some studies have demonstrated that individuals exposed to pesticides and PCBs may have a thinner endometrial lining (85, 86), making implantation more difficult (20, 79, 86, 87). Also, BPA has been associated with an increase in aneuploid oocytes, which can lead to miscarriage (86, 88). A link with miscarriage has also been observed for phthalates, bisphenols, benzophenones, and PCBs (85–89).

### 4.3 Potential transgenerational effects on offspring health and fertility

A large body of research has demonstrated that six major classes of EDCs – phthalates, phenols, perfluorinated compounds, flame retardants, PCBs, and organochlorine pesticides – can cross the placental barrier (5, 6, 90). Despite this, few researchers have investigated the link between prenatal exposure to EDCs and potential adverse effects on fetal development, immediate postnatal health, and fertility. Several studies in mice have observed a correlation between prenatal exposure to DEHP and BPA and the effects on future generations (91, 92). Data suggest that prenatal and ancestral exposure to DEHP negatively impacts reproductive outcomes in F1-F3 generations of mice, by accelerating puberty, disrupting estrous cycle, and decreasing pregnancy rate and fertility index (91). Likewise, studies of *in utero* bisphenol treatment in mice investigated the negative outcomes of EDC exposure such as in three subsequent generations. Their findings evidence that this EDC can influence pubertal onset and reproductive capacity throughout several generations (93, 94).

Fewer publications are available on transgenerational effects in humans. Urinary BPA and BPS concentrations in mothers prior to conception have been shown to be associated to an increased risk of premature birth (95). Few, if any, studies have examined the effects of prenatal EDCs exposure in human offspring. The limited research available focuses on the effects of prenatal exposure on neonatal health. Other studies have shown a negative correlation between prenatal BPA exposure and gestational age (95), while others have investigated a correlation with an increased incidence of systemic anomalies. In particular, prenatal exposure to phthalates appears to be associated with a reduced ano-genital distance in males, an association that does not seem to be confirmed in females (96). No clear correlations have been found between phthalate exposure and cryptorchidism (97), and controversial results have also been found for hypospadias (98, 99).

Further research is needed to determine whether there is a threshold of prenatal exposure above which the harmful effects of EDCs on offspring manifest. More studies are also required to understand the mechanisms by which EDCs influence offspring reproductive health and whether epigenetic mechanisms may be involved in the transmission.

A summary of the effects and outcomes of endocrine-disrupting chemicals in the follicular fluid of women undergoing ART is presented in **Table 2**.

**Table 2:** Human studies on the effects/associations of endocrine-disrupting chemicals in follicular fluid.

EDCs	Study group	Outcome	Reference
EDCs mixture (parabens, phenols, phthalates, PFAS)	Women undergoing ART	Diminished ovarian reserve	(84)
EDCs mixture (bisphenols, parabens, phthalate metabolites, PFAS)	Women undergoing ART	Lower ovarian sensitivity index	(83)
Persistent halogenated organic pollutants (PBDEs and PCBs)	Women undergoing IVF	Longer time to pregnancy and significantly reduced fecundability odds ratios	(78)
Organochlorine pesticides (CB, p,p′ -DDE, HCB, b-HCH)	Women undergoing ART	Reduced fertilization rate and less high-quality embryo during IVF	(20)
Pesticides, PCBs	Women undergoing ICSI	Thinner endometrial thickness	(85)
PCB 28 and 180, pretilachlor, b-cyfluthrin		Lower ovarian retrieval, fertilization, and embryo cleavage rates	
PCB 52		Early pregnancy loss	
PBDEs		Reduced fertilization rate	(79)
p,p’-DDE	Women undergoing IVF	Reduced fertilization rate	(77)
PFAAs	Women undergoing ART	More high-quality embryo during IVF	(21)
	Women undergoing ART (IVF/ICSI) with PCOS	Menstrual irregularity	(66)
	Women undergoing IVF	Less high-quality embryos	(74)
Phthalates and its metabolites	Women undergoing ICSI	Decreased fertilization rate by affecting blastocyst quality	(80)
	Women undergoing ART (IVF/ICSI)	A 10% reduction is observed in the numbers of retrieved oocytes, mature oocytes, and 2PN zygotes, showing an inverse association.	(75)
		Reduced antral follicle count and chance of successful fertility treatment outcomes	(89)
	Women undergoing ART	Poor ovarian reserve	(82)
Phthalate metabolites and phenolic substances	Women undergoing ART	Increased estradiol levels	(19)
Nonylphenols	Women undergoing ICSI	Reduced maturation and fertilization rate	(79)
Benzophenones	Women undergoing ART (IVF/ICSI)	Reduced antral follicle count and chance of successful fertility treatment outcomes	(89)
Bisphenols	Women undergoing ART (IVF/ICSI) with PCOS	Decreased oocytes maturation, number of oocytes retrieved; Decreased aromatase expression and estradiol synthesis in cultured granulosa cells after *in-vitro*	(63)
	Women undergoing ART	Higher counts of oocytes	(76)
		Decreased ability of oocytes to successfully complete meiotic maturation, decreased quality of porcine oocytes	(86)

ART, Assisted Reproduction Techniques; IVF, in vitro fertilization; ICSI, intracytoplasmic sperm injection; CB, chlorinated biphenyl; DDE, dichlorodiphenyldichloroethylene; HCB, hexachlorobenzene; HCH, hexachlorocyclohexane; PBDEs, polybrominated diphenyl ethers; PFAAs, Perfluorinated alkyl acids; PCBs, polychlorinated biphenyls.

## 5 Conclusions and further perspectives

A good number of studies have suggested that EDCs are found in the ovary and FF. Mainly, studies in animals have proven that exposure is harmful and is affecting negatively human fertility, ovarian reserve, and function, and exposure to mixtures seems to worsen these effects. EDCs may alter mitochondrial morphology and function, epigenetics, activate oxidative stress, apoptosis and disrupt hormonal signaling in ovaries and FF. However, current knowledge on this topic is yet scarce, particularly in humans. Further studies are urgently required to investigate the causes of diseases and the mechanisms implicatedUnderstanding how EDCs alter FF composition and function could offer crucial insights into the mechanisms driving reproductive disorders, like PCOS, endometriosis, and infertility. Developing this knowledge is crucial not only for mitigating these adverse effects but also for exploring ways to reverse EDC-induced epigenetic changes, potentially leading to improved reproductive outcomes. *In vitro* mechanistic and *in vivo* studies with the aid of omics are required. Addressing the gaps is essential for developing more effective regulatory strategies and interventions to safeguard female reproductive function against the deleterious effects of EDC exposure.
